# Production and Characterization of the Third-Generation Oxide Nanotubes on Ti-13Zr-13Nb Alloy

**DOI:** 10.3390/ma15062321

**Published:** 2022-03-21

**Authors:** Bożena Łosiewicz, Sandra Skwarek, Agnieszka Stróż, Patrycja Osak, Karolina Dudek, Julian Kubisztal, Joanna Maszybrocka

**Affiliations:** 1 Faculty of Science and Technology, Institute of Materials Engineering, University of Silesia in Katowice, 75 Pułku Piechoty 1A, 41-500 Chorzów, Poland; sandraskwarek99@gmail.com (S.S.); agnieszka.stroz@us.edu.pl (A.S.); patrycja.osak@us.edu.pl (P.O.); julian.kubisztal@us.edu.pl (J.K.); joanna.maszybrocka@us.edu.pl (J.M.); 2 Refractory Materials Division, Institute of Ceramics and Building Materials, Łukasiewicz Research Network, Toszecka 99, 44-100 Gliwice, Poland; k.dudek@icimb.pl

**Keywords:** anodizing, biomaterials, corrosion resistance, oxide nanotubes, Ti-13Zr-13Nb alloy

## Abstract

In the group of vanadium-free titanium alloys used for applications for long-term implants, the Ti-13Zr-13Nb alloy has recently been proposed. The production of a porous layer of oxide nanotubes (ONTs) with a wide range of geometries and lengths on the Ti-13Zr-13Nb alloy surface can increase its osteoinductive properties and enable intelligent drug delivery. This work concerns developing a method of electrochemical modification of the Ti-13Zr-13Nb alloy surface to obtain third-generation ONTs. The effect of the anodizing voltage on the microstructure and thickness of the obtained oxide layers was conducted in 1 M C_2_H_6_O_2_ + 4 wt% NH_4_F electrolyte in the voltage range 5–35 V for 120 min at room temperature. The obtained third-generation ONTs were characterized using SEM, EDS, SKP, and 2D roughness profiles methods. The preliminary assessment of corrosion resistance carried out in accelerated corrosion tests in the artificial atmosphere showed the high quality of the newly developed ONTs and the slight influence of neutral salt spray on their micromechanical properties.

## 1. Introduction

The long-term implant market mainly includes the commercially pure titanium (Cp Ti) and Ti-6Al-4V two-phase (α + β) alloy [1,2,3,4,5,6,7,8,9]. However, the use of toxic alloying additives such as Al and V has been shown to pose a health risk to patients due to their harmful properties, including the induction of allergies, inflammation, Alzheimer’s disease, neuropathy, and more [4,10]. Titanium-based implants containing toxic vanadium are gradually being replaced by vanadium-free titanium alloys containing more biocompatible alloying additives in the form of Mo, Nb, Zr, and Ta, which can also stabilize the β structure in titanium [11,12,13,14,15].

In the group of vanadium-free titanium alloys for long-term implants, the Ti-13Zr-13Nb two-phase (α + β) alloy has recently been proposed, which reveals the self-passivation ability, high biocompatibility, and corrosion resistance in the human body [5,15]. The use of alloying elements such as Nb and Zr influences the stabilization of the β phase and the reduction of Young’s modulus, preventing the shielding effect [4]. The elastic modulus for the Ti-13Zr-13Nb alloy of 79 GPa is closer to that for the bone with a viscoelastic composite microstructure (10–30 GPa) compared to the Ti-6Al-4V alloy (110 GPa) [5]. The mechanical properties of the Ti-13Zr-13Nb alloy and oxide nanotubes formed on its surface by anodizing can be characterized using the nanoindentation method [16,17]. Specifically, knowledge about Young’s modulus to resist deformation should be important to the intelligent drug delivery systems. However, there are limitations to the use of the heat treatment of Ti-13Nb-13Zr alloy through the capability aging (CA) treatment recommended by the ASTM standard, which allows for the evolution of the microstructure and changes in mechanical compatibility [18]. Mechanical strength and Young’s modulus increase with increasing CA time, whereas ductility decreases due to the decomposition of α′ martensite into the (α + β) structure, especially with hard α precipitates. The authors suggest the intrinsic limit of the static heat treatment of the Ti-13Zr-13Nb alloy, which is a disadvantageous phenomenon for biomedical applications requiring high strength and a low Young’s modulus. Thus, other processing methods are sought that can provide mechanical reinforcement without precipitation of the hard α phase.

Currently, intensive research is carried out to develop innovative surface modification methods of the biomedical Ti-13Zr-13Nb alloy to increase its bioactivity, biocompatibility, and long-term stability [15,16,17,18,19,20,21,22,23,24,25,26,27,28,29,30,31,32,33,34,35,36,37,38,39,40,41]. Electrochemical modification can carry out additional functionalization of the Ti-13Zr-13Nb alloy surface, increasing its osteoinductive properties for applications in regenerative medicine and intelligent drug delivery systems [29]. Innovative methods of obtaining multifunctional chitosan-based coatings on the smooth and porous surface of the Ti-13Zr-13Nb alloy using the electrophoretic deposition method (EPD) have been developed [18]. It has been reported that composite chitosan-copper nanoparticles obtained by a one-step EPD with the addition of a dispersing agent reveal strong adhesion, high corrosion resistance, and improved mechanical and antimicrobial properties [20]. Composite chitosan-nanosilver coatings obtained by cataphoretic deposition are characterized by increased surface bioactivity and antibacterial properties [21]. Many efforts have also been made by Barański and co-authors towards the development of EPD technology for the production of nanohydroxyapatite-based coatings with a chemical composition similar to that of the surrounding tissues and showing antibacterial activity [22,23,24,25,26,27]. The addition of nanosilver and nanocopper dispersed in the nanohydroxyapatite coatings on the Ti-13Zr-13Nb substrate increases corrosion resistance and hydrophilicity. Both nanometals together effectively kill bacteria and inhibit biofilm growth. The nanocopper improves the mechanical stability of nanohydroxyapatite coatings. Bioactive calcium-phosphate coatings containing oxide layers formed during the micro-arc oxidation process on the Ti-13Zr-13Nb alloy produced by the selective laser melting are also obtained [28,29]. The porous Ti-13Zr-13Nb scaffold with designed porosity and no harmful effects is also developed using powder metallurgy with and without space holders and SLM methods. Electrochemical oxidation, gaseous oxidation, and chemical oxidation, as well as hydroxyapatite deposition, are applied to its surface modification [30]. The obtained coatings of high-performance structures can support the regeneration process by stimulating the reconstruction of the tissues surrounding the implant, limiting the occurrence of inflammation and of the risk of releasing harmful metal ions from the implant surface to the body. They can also be a source of tissue-forming elements and act as a carrier of medicinal substances.

A self-passive oxide layer on the Ti-13Zr-13Nb alloy surface and its electrochemical properties play an essential role in the long-term implantation [15]. The biocompatible properties of the Ti-13Nb-13Zr alloy can be additionally improved by creating a porous oxide layer on its surface using electrochemical methods [31,32,33,34,35,36,37,38,39,40,41]. Obtaining a porous surface of the Ti-13Zr-13Nb alloy enables the development of innovative long-term implants with increased osteoinductive properties. Recently, it was reported that the oxide nanotubes (ONTs) layer on the surface of titanium and its alloys could also be a carrier for drugs delivered to a specific place and enable their controlled release into the body at a specific rate, depending on the size of the nanotubes [12]. Due to their eluting properties, the ONTs can act as micro- or nano-syringes filled with medicinal, bactericidal, or tissue-forming substances. Electrochemical modification of the Ti-13Zr-13Nb alloy surface may significantly improve the effectiveness of implantological treatment. For this reason, Ossowska and co-authors obtained thin hybrid oxide coatings with a crystalline and nanotubular structure, which were on the Ti-13Zr-13Nb alloy in two-stage oxidation consisting of thermal and electrochemical oxidation processes [31,32]. Oxide layers on the Ti-13Zr-13Nb substrate formed in two-stage anodization in phosphoric acid (first stage) and the presence of hydrofluoric acid (second stage) were also the subject of their research [33]. An attempt was made to oxidize the solid and porous Ti-13Zr-13Nb alloy in H_3_PO_4_ with the addition of HF to form high corrosion-resistant nanotubular layers [34,35]. The ONTs with controlled morphology, length, and diameter on the Ti-13Nb-13Zr alloy were also obtained by anodizing room temperature in hydrofluoric acid solution [36], in (NH_4_)_2_SO_4_ + NH_4_F solution [37,38,39,40], and in ethylene glycol solution with the addition of NH_4_F [41].

Preliminary research has shown that it is possible to produce biocompatible third-generation ONTs on the surface of the Ti-13Nb-13Zr alloy in a 1 M ethylene glycol solution with the addition of 4% NH_4_F by anodizing at 50 V for 80 min at room temperature [41]. The results of structural and microscopic studies confirmed the possibility of obtaining single-wall oxide nanotubes (SWNTs) under the proposed electrochemical oxidation conditions. The morphological parameters of the obtained amorphous SWNTs were determined, such as the length (~10 μm) and the external (362 nm) and internal (218 nm) diameter of the nanotube. The formed nanotubes consisted of oxides of alloying elements, such as TiO_2_, Nb_2_O_5_, ZrO_2_, and ZrO_x_. It was revealed that the hemolysis of the Ti-13Nb-13Zr alloy was eliminated, and its ability to osseointegrate was increased by forming an amorphous layer of SWNTs. The quantitative characteristics of the kinetics of the corrosion process of the obtained SWNTs with the simultaneous characterization of the capacitive properties of the system were carried out using the electrochemical impedance spectroscopy method, which showed a slight decrease in its corrosion resistance in saline solution compared to the Ti-13Zr-13Nb alloy.

The present work continues preliminary research on the functionalization of the Ti-13Zr-13Nb alloy surface by obtaining the third-generation SWNTs to develop innovative long-term implants [41]. In this work, new voltage–time conditions for SWNT production on the Ti-13Zr-13Nb alloy by anodizing in a 1 M ethylene glycol solution with the addition of 4% NH_4_F are proposed, which have not been described in the literature. For the first time, a wide range of anodizing voltages from 5 to 35 V for 2 h was used in 1 M C_2_H_6_O_2_ + 4 wt% NH_4_F electrolyte. The increased concentration of F^−^ ions, up to 4 wt%, was aimed at increasing their activity in the anodizing process. The physicochemical characteristics of the obtained SWNTs included research on their microstructure, chemical composition, thickness, contact potential difference, and surface roughness. The long-term corrosion resistance of the newly obtained SWNTs and the comparative Ti-13Zr-13Nb substrate to the effect of neutral salt spray (NSS) in a salt chamber was tested. The influence of NSS on the micromechanical properties of the tested materials was assessed in the microhardness measurements.

## 2. Materials and Methods

### 2.1. Substrate Preparation

The substrate was Ti-13Zr-13Nb (wt%) alloy (BIMO TECH, Wrocław, Poland) in 5 mm thick discs with a 15 mm radius. The chemical, mechanical, and metallurgical requirements for wrought Ti-13Zr-13Nb alloy bars and wires to be used in the manufacture of surgical implants are described in ASTM F1713-08(2021)e1 standard [42]. A mirror-like surface of the samples was obtained with a polishing machine at 250 rpm using silicon carbide (SiC) abrasive papers of 600, 1200, 3000, and 5000 gradations (Struers Inc., Cleveland, OH, USA). The polished samples were cleaned in two stages in an ultrasonic bath with acetone (Avantor Performance Materials Poland S.A., Gliwice, Poland) and then with ultrapure water (Milli-Q Advantage A10 Water Purification System, Millipore SAS, Molsheim, France) for 20 min each at room temperature.

### 2.2. Production of SWNTs on Ti-13Zr-13Nb Alloy

The method of preparing the electrodes is described in detail in an earlier work [12]. To remove oxides from the Ti-13Zr-13Nb alloy surface, immediately before anodizing, each electrode with a geometric surface area of 3.14 cm^2^ was immersed in 25% *v*/*v* HNO_3_ (Avantor Performance Materials Poland S.A., Gliwice, Poland) for 10 min at room temperature and then re-cleaned with Milli-Q water in an ultrasonic bath for 20 min at room temperature.

The SWNTs on the Ti-13Zr-13Nb alloy were produced by one-step anodizing in 1 M C_2_H_6_O_2_ solution with 4 wt% content of NH_4_F at room temperature using a PWR800H high-current power supply (Kikusui Electronics Corporation, Yokohama, Japan). Ethylene glycol (anhydrous, 99.8%) and ammonium fluoride (≥99.99% trace metals basis) were supplied by Sigma-Aldrich (Saint Louis, MO, USA). The electrochemical oxidation was carried out at a voltage (U) in the range of 5–35 V with step 5 V and a time (t) for 120 min. A two-electrode system that was used is described in [38]. After anodizing was completed, each anode was placed in vigorously stirred Milli-Q water for 5 min.

### 2.3. Physicochemical Characteristics of SWNTs on Ti-13Zr-13Nb Alloy

To determine the microstructure of the Ti-13Zr-13Nb alloy before and after anodizing, the TESCAN Mira 3 LMU scanning electron microscope (SEM, TESCAN ORSAY HOLDING, Brno-Kohoutovice, Czech Republic) was used. Images were collected by secondary electrons (SE). The measurements were carried out on the samples covered by a chromium layer using Quorum Q150T ES equipment (Quorum Technologies, East Sussex, UK). Chemical composition was analysed using SEM combined with an Energy Dispersive Spectrometer (EDS, Oxford Instruments, Abingdon, UK).

The scanning Kelvin probe (SKP) method was used to determine the thickness of the obtained SWNTs and their local contact potential difference (V_CPD_) in the air using the PAR Model 370 Scanning Electrochemical Workstation (Princeton Applied Research, Oak Ridge, TN, USA). The tip of the tungsten microprobe was held above the sample surface at a distance of approx. 100 µm. The SWNTs layers’ thickness measurements were carried out in a linear scan mode over a 4000 μm-long section, including the exposed substrate and the produced oxide layer. To determine the V_CPD_, an area of 1000 × 1000 μm^2^ was scanned. The tip–sample system was considered a capacitor, and the V_CPD_ was determined from the difference of the work function for the tested sample and the tip, as described in [6].

The surface roughness of the Ti-13Zr-13Nb alloy before and after anodizing was studied by the Mitutoyo Surftest SJ-500/P profilometer (Mitutoyo Corporation, Kanagawa, Japan). To measure changes in the surface profile, a measuring step of 0.1 μm and a speed of 200 μm s^−1^, over a length of approx. 5 mm, were used. The surface texture parameters were registered according to ISO 4287 [43]. The processing and development of the recorded parameters were carried out using the FORMTRACEPAK computer program.

### 2.4. Corrosion Test of SWNTs on Ti-13Zr-13Nb Alloy in Artificial Atmosphere

**Figure 1:**
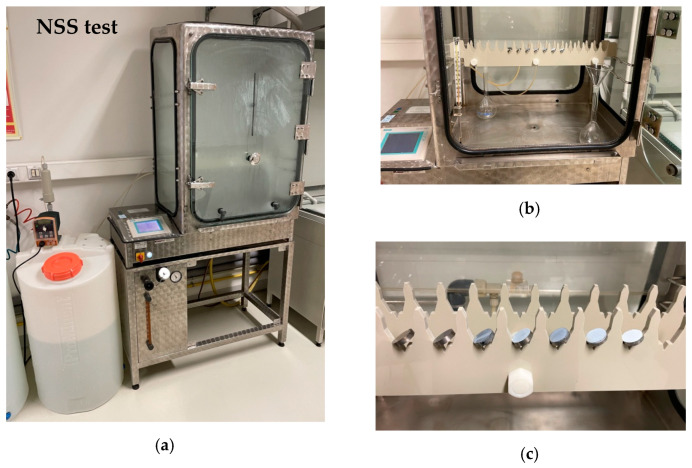
The HKS 400 salt spray chamber during the NSS test according to ISO 9227:2017 [44]: (**a**) Arrangement of collecting devices in the salt chamber; (**b**) Method of mounting the test samples in the holder (**c**).

The SWNTs on the Ti-13Zr-13Nb alloy were tested according to ISO 9227:2017 in the NSS test [44]. The universal HKS 400 salt spray chamber (KÖHLER Automobiltechnik GmbH, Lippstadt, Germany) during the NSS test is shown in Figure 1a. Sodium chloride of recognized analytical grade (Avantor Performance Materials Poland S.A., Gliwice, Poland) and Milli-Q water were used to prepare a 5% NaCl solution. 

Two collecting devices were placed in the salt chamber, consisting of glass funnels with feet inserted into flat-bottomed glass flasks with volumes of 500 cm^3^ (Figure 1b). Funnels with a diameter of 100 mm were used with a collection area of approx. 80 cm^2^. The collecting devices were located in the chamber where the test specimens were placed, one near the spray inlet and one away from the inlet. The collecting devices were positioned in such a way that only salt spray was collected and not liquid dripping from the samples or the chamber components.

Before starting the NSS test, the samples were acclimatized for 24 h at a temperature of 25(2) °C and a relative air humidity of 50(5)%. The specimens were placed face up at an angle of 20(5) °C to the vertical in the NSS-resistant holders to prevent the salt spray from falling directly onto the test specimens (Figure 1c).

The operating parameters of the salt chamber are presented in Table 1. The extended uncertainty of the determined parameters was estimated for a confidence level of about 95%. 

After removing the salt chamber, the samples were gently washed with Milli-Q water and air-dried for 24 h. The surface condition of the samples, after the NSS test, was assessed visually.

### 2.5. Micromechanical Properties of SWNTs on Ti-13Zr-13Nb Alloy

The microhardness of the tested samples was measured before and after the NSS test by the Vickers method with a hardness scale of HV = 0.1 using a Wilson^®^-WolpertTM Microindentation Tester 401MVD (Wilson Instruments, LLC, Carthage, TX, USA). A regular, quadrilateral diamond pyramid with a dihedral angle α = 136° was used as the indenter under load F perpendicular to the tested sample surface. According to the ISO 6507-1 standard [45], the result of the measurement was the diagonal of the obtained square imprint. The method for checking and calibrating the hardness testers and the diagonal of the measuring system used for the Vickers hardness measurement is available in ISO 6507-2 [46]. The calibration method of reference standards for the indirect checking of Vickers hardness testers for indentations ≥0.020 mm is described in ISO 6507-3 [47].

## 3. Results and Discussion

### 3.1. Formation of SWNTs on Ti-13Zr-13Nb Alloy

**Figure 2:**
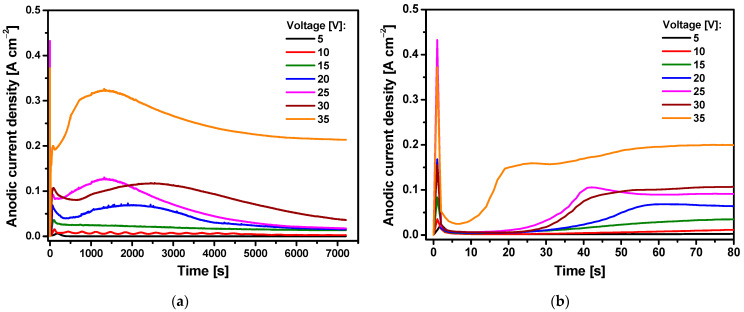
Current transients for the Ti-13Zr-13Nb electrode in 1 M C_2_H_6_O_2_ + 4 wt% NH_4_F electrolyte during anodizing for: (**a**) 7200 s; (**b**) first 80 s.

The current transients corresponding to the anodizing of Ti-13Zr-13Nb electrode in fresh electrolyte of 1 M C_2_H_6_O_2_ containing 4 wt% NH_4_F are shown in Figure 2. It can be observed that an increase in the anodizing potential causes an increase in the current density recorded during the electrochemical oxidation process. The shape of the obtained curves is not similar to the current transients observed in the literature for forming ordered titania nanotubes in an ethylene glycol electrolyte without and with the addition of 0.13–1 wt% water in the presence of fluoride ions [48]. In Figure 2, the behavior of the anodic current density as a function of time shows similarity to the current transient observed for the Ti-13Zr-13Nb electrode during anodizing in 0.5 wt% HF [36] and in 1 M (NH_4_)_2_SO_4_ + 0.5 wt% NH_4_F [37] electrolytes. The typical initial decay and the increase in the anodic current density before reaching the quasi-steady-state value are observed in 1 M C_2_H_6_O_2_ electrolyte containing 4 wt% NH_4_F. The inset of Figure 2b for the first 80 s of anodizing shows that the quasi-steady-state conditions are achieved more quickly with increasing anodizing voltage.

Such a course of current transients is related to various stages of the process of pore formation on the surface of the Ti-13Zr-13Nb alloy. In the first anodizing stage, an oxide layer with strong barrier properties is created, the presence of which is evidenced by a decrease in the anodic current density. In the next stage, the oxide surface is locally activated due to the activity of fluoride ions, and the pores formed randomly increase their size. As a result of the growth of pores, the active surface is increased, which causes an increase in the anodic current density. In the last stage, the resulting pores share the available current evenly among themselves, and the process of self-assembly under steady-state conditions takes place.

SWNTs can be produced electrochemically on the surface of the Ti-13Zr-13Nb electrode in 1 M C_2_H_6_O_2_ electrolyte in the presence of F^−^ ions, which can form water-soluble complexes with Ti, Zr, and Nb according to the Reactions (1)–(3) [11,12,49,50]:

(1) $${\mathrm{TiO}}_{2}+6F^{-}+6H^{+}\to {\left[{\mathrm{TiF}}_{6}\right]}^{2-}+2H_{2}O$$

(2) $${\mathrm{ZrO}}_{2}+6F^{-}+6H^{+}\to {\left[{\mathrm{ZrF}}_{6}\right]}^{2-}+2H_{2}O$$

(3) $${\mathrm{Nb}}_{2}O_{5}+12F^{-}+10H^{+}\to 2{\left[{\mathrm{NbF}}_{6}\right]}^{-}+5H_{2}O$$

Under the influence of the electric field, the fluoride ions migrate deep into the formed oxide layer. F^−^ ions compete with O^2−^ ions and cause the local dissolution of the oxide layer. Simultaneously, the fluoride ions inhibit the deposition of titanium hydroxides on the oxide layer. Reaction (4) describes the complexation of Ti^4+^ ions, which migrate to the oxide surface and are ejected at the interface [12,49,50]:

(4) $${\mathrm{Ti}}^{4+}+6F^{-}\to {\left[{\mathrm{TiF}}_{6}\right]}^{2-}$$

The release of oxygen from water occurs according to Reaction (5) [12,49,50]:

(5) $$2H_{2}O+{4e}^{-}\to O_{2}+{4H}^{+}$$

A detailed mechanism of SWNTs formation on titanium and its alloys has been described previously in [12,49,50].

### 3.2. SEM Study of Microstructure

**Figure 3:**
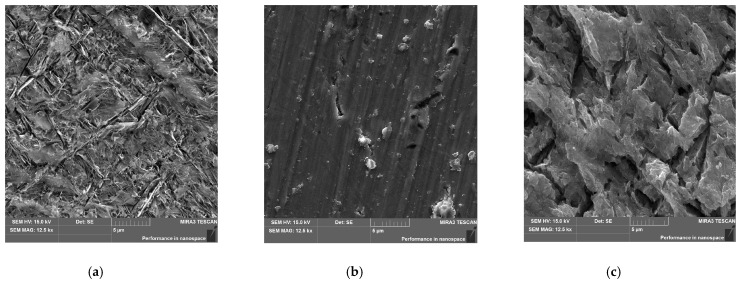
SEM image of the microstructure of the Ti-13Zr-13Nb alloy before and after anodizing in 1 M C_2_H_6_O_2_ + 4 wt% NH_4_F electrolyte: (**a**) Etched; (**b**) Anodized at 5 V for 120 min; (**c**) Anodized at 10 V for 120 min.

Figure 3a shows an SEM image of the microstructure of the biomedical Ti-13Nb-13Zr alloy before anodizing. The surface of this bi-phase alloy composed of a mixture of α and β phases was previously etched for a few seconds in the Kroll’s reagent consisting of Milli-Q water, hydrofluoric acid, and nitric acid [41]. One can see the typical acicular martensitic α′ laths arranged in various directions and embedded in the β matrix [33].

The microstructure of the Ti-13Zr-13Nb alloy after anodizing in 1 M C_2_H_6_O_2_ + 4 wt% NH_4_F electrolyte at the lowest voltage of 5 V reveals the presence of the oxide layer, which is characterized by a lack of the nanotubular structure despite the long anodizing time of 120 min (Figure 3b). The poorly developed surface morphology of the thin oxide layer with fine microcracks is visible, reflecting the structure of the polished substrate. Increasing the anodizing voltage to 10 V resulted in obtaining a porous surface of the oxide layer, showing intense cracking and greater thickness (Figure 3c). However, the presence of oxide nanotubes is still not observed.

**Figure 4:**
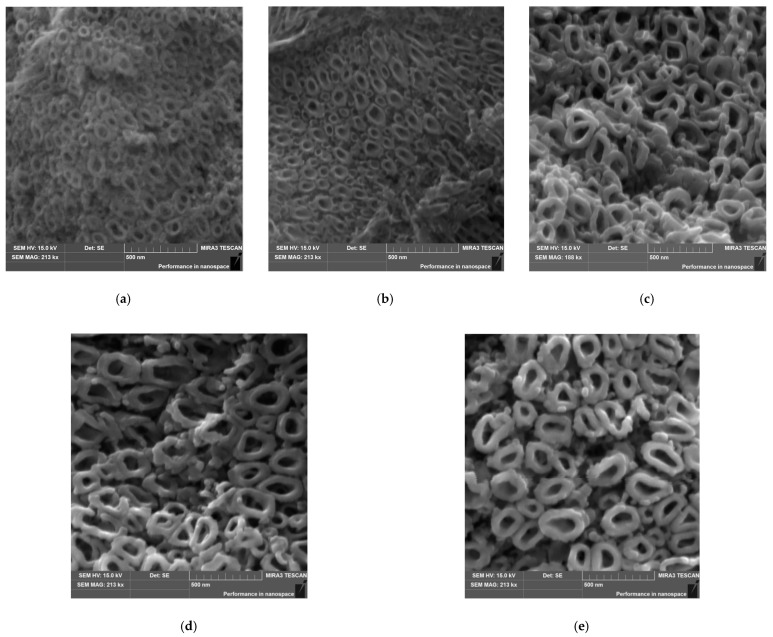
SEM image of the microstructure of SWNTs layer formed on the Ti-13Zr-13Nb alloy in 1 M C_2_H_6_O_2_ + 4 wt% NH_4_F electrolyte under anodizing conditions: (**a**) 15 V for 120 min; (**b**) 20 V for 120 min; (**c**) 25 V for 120 min; (**d**) 30 V for 120 min; (**e**) 35 V for 120 min.

The SEM images of an on-top general view of the Ti-13Zr-13Nb alloy anodized at voltages within the range of 15 to 35 V for 120 min present the microstructure of oxide layers with a parallel arrangement of the SWNTs (Figure 4). The SWNTs with an elliptical cross-sectional shape and smooth walls are evenly distributed over the surface of the oxide layers. The diameter of the obtained third-generation oxide nanotubes and their order increase with the anodizing voltage.

**Figure 5:**
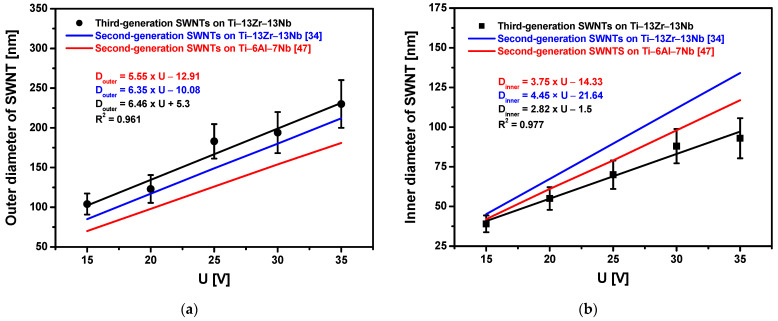
The oxide nanotube diameter on the Ti-13Zr-13Nb alloy surface as a function of anodizing voltage (U) for 120 min in 1 M C_2_H_6_O_2_ + 4 wt% NH_4_F electrolyte: (**a**) Outer diameter (D_outer_); (**b**) Inner diameter (D_i_).

Based on the SEM images of the microstructure, it was found that the outer diameter (D_outer_) varied from 104(13) nm, for the Ti-13Zr-13Nb alloy anodized at 15 V, to 230(30) nm, for the sample oxidized at 35 V (Figure 5). The inner diameter (D_inner_) changed from 39(5) to 93(13) nm. Both D_outer_ and D_inner_ increased linearly with increasing anodizing voltages, with more than twice the growth rate observed for D_outer_. The linear equations describing the change of the SWNTs diameters as a function of the anodizing voltage are presented in Figure 5. Comparing these equations with the equations determined for second-generation SWNTs on the Ti-13Zr-13Nb alloy [37] and second-generation SWNTs on the Ti-6Al-7Nb alloy [50] obtained by anodizing for 120 min in 1 M (NH_4_)_2_SO_4_ + 0.5 wt% NH_4_F shows that D_outer_ growth is the fastest for third-generation SWNTs on the Ti-13Zr-13Nb alloy in 1 M C_2_H_6_O_2_ + 4 wt% NH_4_F electrolyte, whereas D_inner_ growth is the slowest. The obtained results indicate that the thickest walls characterize third-generation SWNTs on the Ti-13Zr-13Nb alloy. The derived linear equations can be used in the future to obtain SWNTs on the Ti-13Zr-13Nb alloy with the assumed morphological parameters (Figure 5).

**Figure 6:**
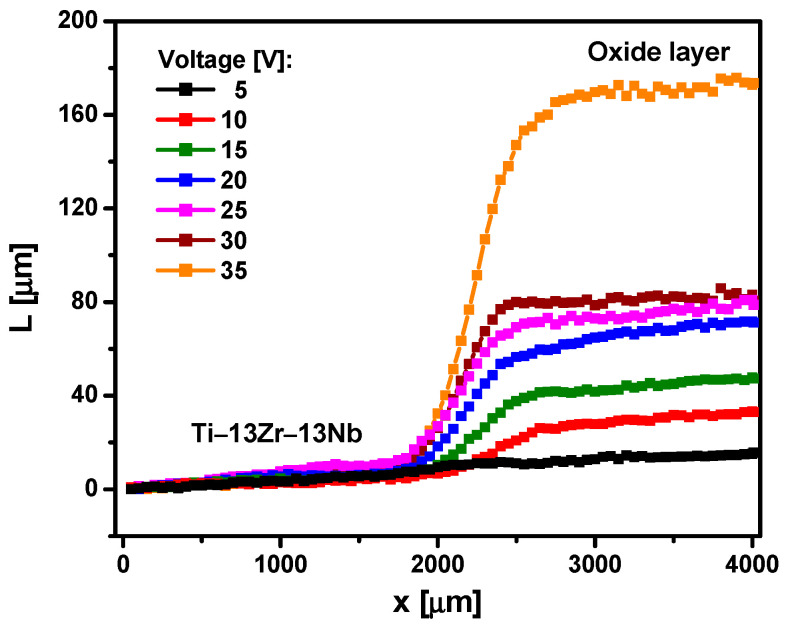
The thickness (L) of the oxide layer on the Ti-13Zr-13Nb alloy surface obtained by anodizing in 1 M C_2_H_6_O_2_ + 4 wt% NH_4_F electrolyte at 5–35 V for 120 min, measured over distance x.

Figure 6 shows the thickness (L) of the oxide layers formed on the Ti-13Zr-13Nb alloy surface formed during anodizing in 1 M C_2_H_6_O_2_ + 4 wt% NH_4_F electrolyte at U of 5–35 V for 120 min. Measurements were made using the scanning Kelvin probe method on a 4000 µm section, which included the area of the Ti-13Zr-13Nb substrate and the area of the oxide layer. The L of the oxide layer on the Ti-13Zr-13Nb substrate varied from 15.64(71) μm, for the sample anodized at U of 5 V, to 167.52(60) μm, for the sample oxidized at U of 35 V. The obtained results indicate that third-generation SWNTs on the Ti-13Zr-13Nb alloy obtained under the proposed anodizing conditions are much longer than first-generation [36] and second-generation [40] SWNTs on the Ti-13Zr-13Nb alloy. In 0.5% HF electrolyte at an anodizing voltage of 20 V for 120 min, we obtained an L of only 500 nm [36]. In 1 M (NH_4_)_2_SO_4_ electrolyte under voltage–time conditions of 20 V for 120 min, the L of SWNTs of 3.9 µm was found [40]. In 1 M C_2_H_6_O_2_ + 4 wt% NH_4_F electrolyte under the same anodizing conditions, the L of SWNTs is 65.25(74) µm (Figure 6). The obtained results show that using the electrolyte based on ethylene glycol allows obtaining a 130-fold and 16-fold increase in L of third-generation SWNTs compared to SWNTs of the first [36] and second [40] generation, respectively.

Based on the obtained results, it can be concluded that the applied electrochemical oxidation voltage and the electrolyte play a key role in tailoring the third-generation SWNTs layers on the Ti-13Zr-13Nb alloy surface. The total surface area (A_total_) of the obtained SWNTs was calculated according to Equation (6) [12,41,51]:

(6) $$A_{\mathrm{total}}=2\pi (D_{\mathrm{outer}}^{2}-D_{\mathrm{inner}}^{2})+2\pi L(D_{\mathrm{outer}}+D_{\mathrm{inner}})$$

**Figure 7:**
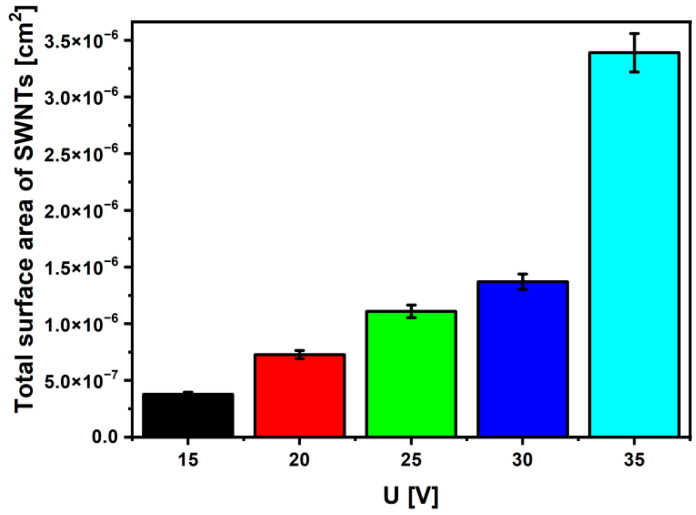
The total surface area (A_total_) of SWNTs on the Ti-13Zr-13Nb alloy surface obtained by anodizing in 1 M C_2_H_6_O_2_ + 4 wt% NH_4_F electrolyte at U of 15–35 V for 120 min.

Figure 7 shows that A_total_ takes the values from 3.78(19) × 10^−7^ cm^2^, for the SWNTs layer on the Ti-13Zr-13Nb alloy anodized at 15 V, to 3.39(17) × 10^−6^ cm^2^, for the SWNTs layer produced on the sample oxidized at 35 V, which proves a 9-fold increase in A_total_ in the tested voltage range. The obtained results indicate that the rise in A_total_ ensures a growth in the contact surface between the oxide layer and the tissue, significantly accelerating the osseointegration process [1,2,3,4,5].

The proposed surface functionalization of the Ti-13Zr-13Nb alloy allows obtaining self-organizing SWNTs layers with a wide range of geometries and lengths. The obtained porous SWNTs show a morphology very similar to bone tissue structures. The new, additionally produced top SWNTs layer is designed to accelerate the osseointegration process, reduce the risk of the releasing of harmful compounds from the implant into the body, and the occurrence of inflammation [15,16,17,18,19,20,21,22,23,25,30,31,32,33,34,35,36,37,38,39,40,48,49]. In line with the latest trends in implantology, the layer of oxide nanotubes can be a carrier for drugs delivered to a specific location and enable their controlled release into the body at a specific rate, depending on the size of the SWNTs [12,29,41]. Oxide nanotubes can also be a carrier for bactericides or tissue-forming substances [23,24,26,27,29].

### 3.3. Chemical Composition of SWNTs on Ti-13Zr-13Nb Alloy

**Figure 8:**
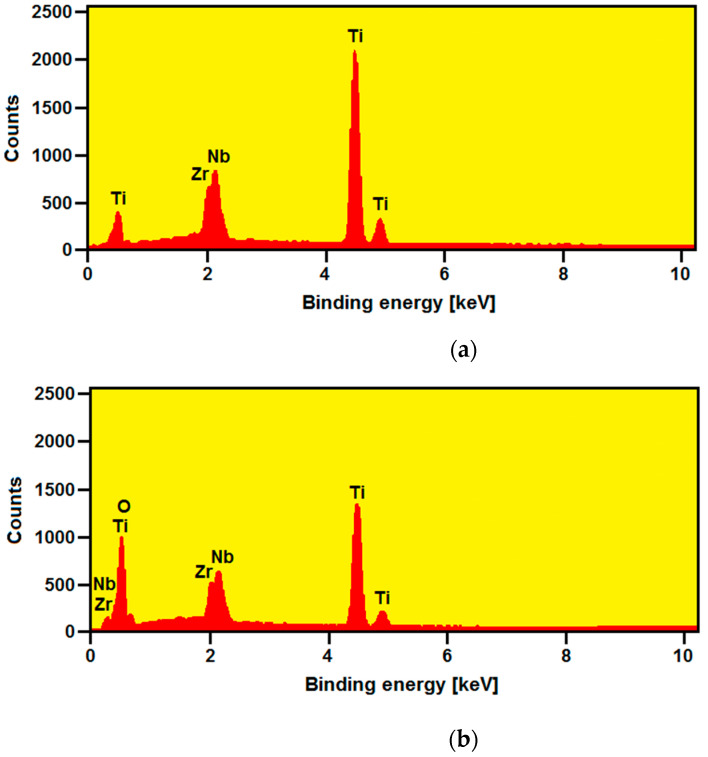
EDS spectrum for the Ti-13Zr-13Nb alloy surface: (**a**) Before anodizing; (**b**) After anodizing at 35 V for 120 min.

The control analysis of the chemical composition of the commercial Ti-13Zr-13Nb alloy before and after anodizing was carried out using the EDS method in selected micro-areas. The EDS spectrum for the substrate before oxidation is shown in Figure 8a.

The obtained relationship between the number of counts and the radiation energy reveals the presence of peaks originating from the metallic components of the substrate, i.e., Ti, Zr, and Nb. Quantitative analysis of the obtained EDS spectrum shows the surface content of the elements in the following amounts (wt%): 74.3(6) for Ti, 12.9(6) for Zr, and 12.8(7) for Nb. These results are in accordance with the chemical composition specified for the bulk Ti-13Zr-13Nb. The assumed chemical composition of the Ti-13Zr-13Nb alloy ensures optimal biocompatible properties and long-term corrosion resistance in a biological environment, which is necessary in medical applications [15,31,33,34,35,36,37,38,39,40,41].

On all EDS spectra obtained for the Ti-13Zr-13Nb alloy after the electrochemical oxidation, oxygen peaks were also observed apart from the alloying component peaks. Figure 8b shows an exemplary EDS spectrum from a micro-area on the surface of the Ti-13Zr-13Nb alloy after anodizing at 35 V for 120 min. The intensity of the oxygen-derived peak increased with the anodizing voltage, resulting in the amorphous SWNTs layer being produced [41].

### 3.4. Electronic Properties of SWNTs on Ti-13Zr-13Nb Alloy

**Figure 9:**
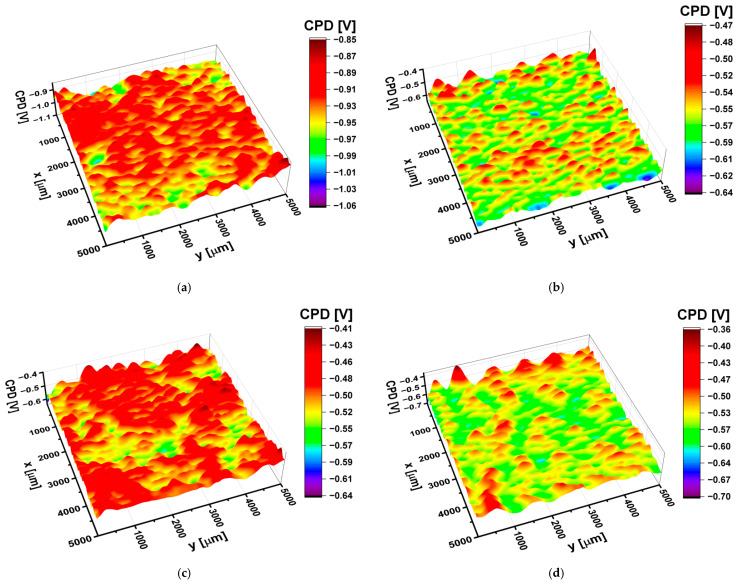
The contact potential difference (CPD) map for the Ti-13Zr-13Nb alloy surface: (**a**) Before anodizing; (**b**) After anodizing at 15 V for 120 min; (**c**) After anodizing at 25 V for 120 min; (**d**) After anodizing at 35 V for 120 min.

The effect of oxidation voltage on the electronic properties of the Ti-13Zr-13Nb alloy was studied using the SKP method. The contact potential difference maps of Ti-13Zr-13Nb alloy before and after oxidation at voltages from 5 V to 35 V are shown in Figure 9.

**Figure 10:**
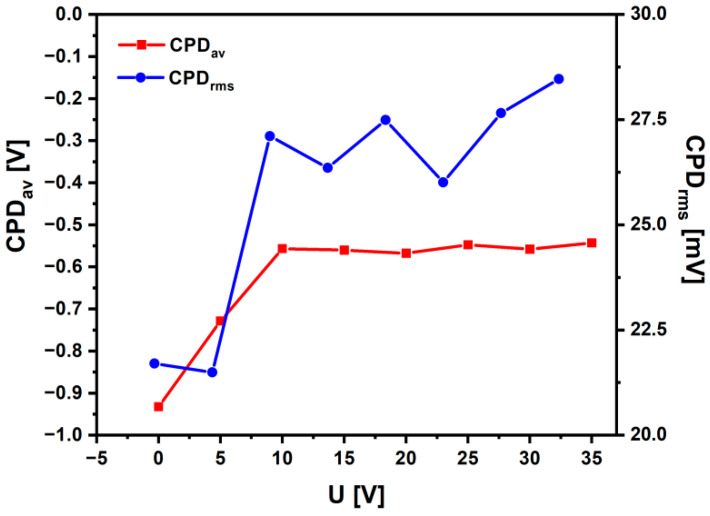
The arithmetic mean (CPD_av_) and root mean square of height irregularities (CPD_rms_) for the Ti-13Zr-13Nb alloy surface before and after anodizing.

Statistical analysis of the obtained CPD maps allowed us to determine the arithmetic mean (CPD_av_), root mean square of height irregularities (CPD_rms_), skewness (CPD_sk_), and kurtosis (CPD_ku_), i.e., parameters that quantitatively determine the electronic properties of the material surface. The obtained dependences of CPD_av_ and CPD_rms_ as a function of the oxidation voltage are shown in Figure 10.

The Ti-13Zr-13Nb alloy at the initial state is characterized by the smallest values of CPD_av_ and CPD_rms_. Oxidation of the alloy surface causes an increase in CPD_av_ and CPD_rms_. In particular, it was found that, up to the value of 10 V, the CPD_av_ increased by about 40%, whereas for the voltage range from 10 to 35 V, the change of the CPD_av_ was about 2%. It has been stated that an increase in the contact potential difference correlates with an increase in the thickness of the oxide coating formed on the Ti-13Zr-13Nb alloy. The oxidation of the Ti-13Zr-13Nb alloy at voltages above 10 V causes a slight increase (ca. 1.2 times) in the CPD_rms_ parameter, indicating only a slight rise in the heights of CPD peaks and valleys. Thus, the surfaces of the oxide coatings formed at oxidation voltages from 5 to 35 V are characterized by similar height irregularities around the average. Generally, skewness and excess kurtosis describe symmetry and shape of the contact potential difference heights. It was found that, with the increase in oxidation voltage, CPD_sk_ increased from −0.3 to 0.3, and CPD_ku_ decreased from 1 to 0.25. Thus, the CPD_sk_ parameter showed a predominance of valleys on the surface of Ti-13Zr-13Nb alloy at the initial state. The oxidation process changes the symmetry of CPD heights, and for coating obtained at 35 V, there is an excess of CPD peaks. In turn, CPD_ku_ indicates the presence of profound valleys on the surface of Ti-13Zr-13Nb alloy at the initial state. These discontinuities disappear during the oxidation of Ti-13Zr-13Nb alloy because they are covered with oxide.

### 3.5. Geometric Structure of the Surface

**Figure 11:**
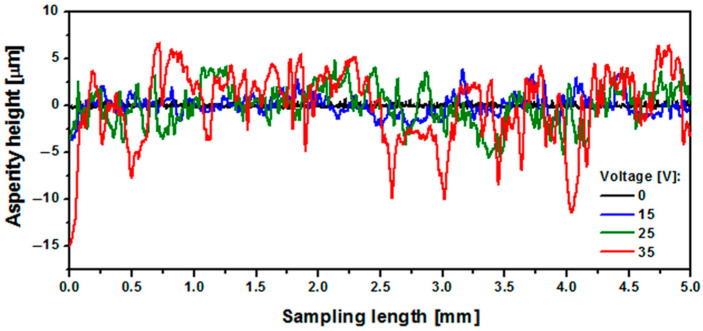
Roughness profile for the Ti-13Zr-13Nb alloy surface before and after anodizing at 15–35 V.

The geometric structure of the surface (GSS) is one of the key factors determining the ability of the surface of biomaterials to osseointegrate [1,2,3,4,5]. The GSS of the Ti-13Zr-13Nb alloy before and after the anodizing was characterized based on surface microgeometry measurements in a two-dimensional (2D) system. The ISO 4287 standard was the basis for determining the basic surface texture parameters [43]. The effect of the anodizing conditions on the GSS of the Ti-13Zr-13Nb alloy was discussed based on selected profile height parameters, such as Ra (arithmetic mean deviation of the roughness profile), Rz (maximum height of the roughness profile), Rp (maximum peak height of the roughness profile), and Rv (maximum valley depth of the roughness profile). Before determining the roughness parameters, the measured profiles were aligned. The analysis was carried out on symmetrical surface profiles. Figure 11 shows the exemplary 2D roughness profile for the Ti-13Zr-13Nb alloy surface before and after anodizing at 15–35 V.

**Figure 12:**
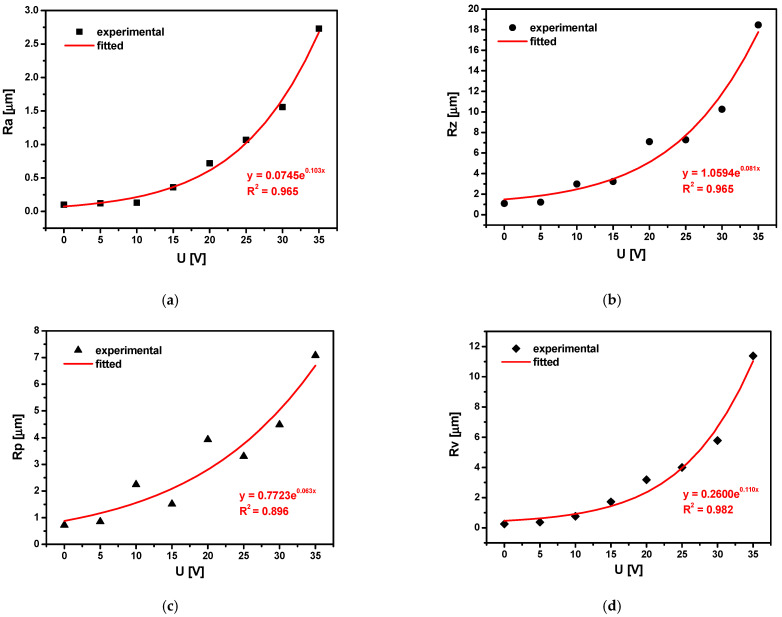
Basic surface texture parameters for the Ti-13Zr-13Nb alloy surface before and after anodizing: (**a**) Ra; (**b**) Rz; (**c**) Rp; (**d**) Rv.

Basic surface texture parameters for the Ti-13Zr-13Nb alloy surface as a function of anodizing voltage are presented in Figure 12.

The experimental data are shown as symbols (black), and the exponential fit is shown as a solid line (red). As a result of surface treatment of the Ti-13Zr-13Nb alloy by anodizing, an exponential increase in Ra (Figure 12a), Rz (Figure 12b), Rp (Figure 12c), and Rv (Figure 12d) was achieved.

At the microscale, no deviations of the roughness profile from the mean line were observed for the non-anodized Ti-13Zr-13Nb surface (Figure 11a). The Ra of 0.10 µm that best recognizes overall surface roughness proves the smooth surface of the alloy before anodizing (Figure 12a). The highest Ra of 2.73 is observed for the surface anodized at 35 V (Figure 12a). The roughness profile for the sample oxidized at the highest anodizing voltage testifies to the most porous surface with numerous pores with a depth of 10 µm, as seen in Figure 11d.

The obtained results indicate the micro-rough surface for which the most suitable Ra is 1 to 3 μm [6,9]. This means that the SWNTs layers produced on the Ti-13Zr-13Nb alloy during the anodizing voltages of 25, 30, and 35 V for 120 min have the optimal Ra. It can be assumed that their surfaces can ensure implant stability, support osseointegration, and reduce the risk of metal ion release due to corrosion processes and tribological wear [1,2,4,5]. The prerequisite for long-term success in implant treatment is, above all, osseointegration, i.e., a direct, structural, and functional connection between the living bone and the implant surface, and the integration of the implant surface with both hard and soft tissues. Bone integration of titanium implants is possible because the oxygen contained in the bone tissue forms a layer of highly biocompatible titanium dioxide on the surface of the titanium implant, on which new, mineralizing bone tissue can be deposited, forming the proper fixation of the implant. The introduction of the intraosseous implant into the bone causes traumatization of the bone tissue, which requires subsequent regeneration. The obtained SWNTs on Ti-13Zr-13Nb alloy meet the necessary condition for the occurrence of osteoinductive properties of the biomaterial, which is the presence of sufficiently large pores with the minimum pore connection size below 50 µm for the ingrowth of blood vessels and transport of cells to the core of the material [52].

### 3.6. Corrosion Resistance of SWNTs on Ti-13Zr-13Nb Alloy in an Artificial Atmosphere 

**Figure 13:**
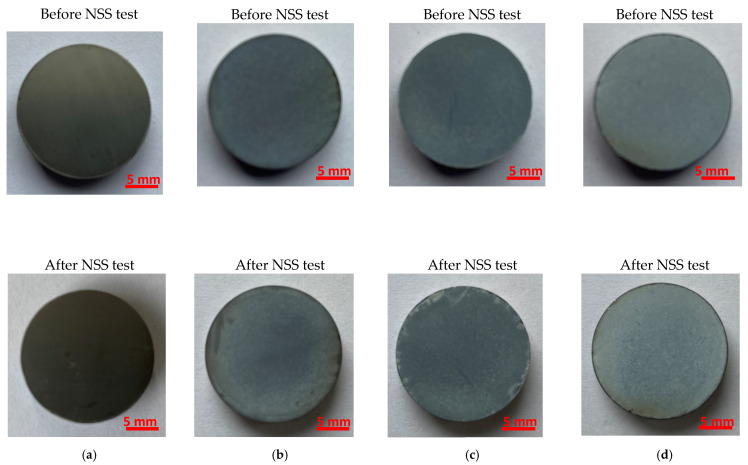
The Ti-13Zr-13Nb alloy surface before and after NSS test according to ISO 9227:2017 [44]: (**a**) Non-anodized substrate; (**b**) After anodizing at 15 V for 120 min; (**c**) After anodizing at 25 V for 120 min; (**d**) After anodizing at 35 V for 120 min.

An assessment of the corrosion resistance of the Ti-13Zr-13Nb alloy before and after anodizing to the effect of neutral salt spray was performed in the NSS test according to ISO 9227:2017 [44]. The surface of exemplary samples before and after exposure to neutral salt spray for 168 h is shown in Figure 13. The qualitative evaluation of the samples on a macro scale using the visual assessment method showed only slight changes in the surface appearance of the samples before and after the NSS test. In the case of the grey non-anodized substrate after the NSS test, a few minor corrosion spots darker in color on the Ti-13Zr-13Nb alloy surface may be related to the surface preparation method and/or related to visible local chemical heterogeneities (Figure 13a). On the surface of the samples, after anodizing at 15 V (Figure 13b), 25 V (Figure 13c), and 35 V (Figure 13d), a slightly lower intensity of the blue color can be observed, which may be caused by the dissolution of the SWNTs layer. At the edges of the samples after the NSS test, discoloration, local breakthroughs to the substrate, and chipping were visible, probably related to the greater brittleness of the SWNTs produced at higher anode current densities at the edges of the samples (edge effects). It is worth adding that the unprotected edges of the samples are particularly exposed to the action of aggressive salt spray and are potential places for the initiation of corrosion damage. The highest quality in accelerated laboratory tests in the neutral salt spray was demonstrated for the Ti-13Zr-13Nb alloy after anodizing at 35 V for 120 min, characterized by the highest SWNTs thickness of 167.52(60) μm (Figure 13d).

The Ti-13Zr-13Nb alloy belongs to the newest class of biomedical alloys. This alloy is highly resistant to corrosion and simultaneously exhibits a low elastic modulus, high strength, and excellent hot and cold workability, thus meeting the stringent requirements for materials used for medical implants [53,54]. The mechanical properties of Ti-13Zr-13Nb alloy can be tailored in a wide range by hot working, cold working, and heat treatment. Microhardness measurements can be used to assess the structural integrity of the bone in the porous implant surface and the bone surrounding the implant in the long-term bone ingrowth process. Aggressive chloride ions present in neutral salt spray can strongly influence the corrosive behavior of Ti-13Zr-13Nb alloy. Therefore, it seems advisable to compare the micromechnical properties of the tested alloy before and after the salt spray test.

**Figure 14:**
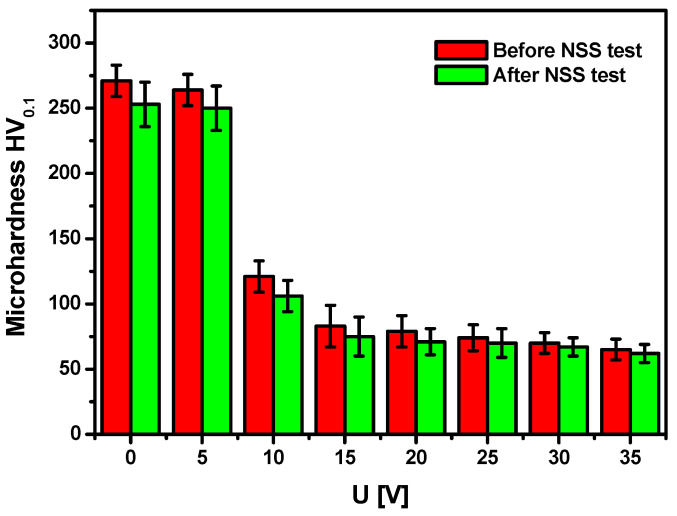
Microhardness of the Ti-13Zr-13Nb alloy surface before and after the NSS test according to ISO 9227:2017 [44] for non-anodized substrate (U = 0 V) and after anodizing at U of 5–35 V for 120 min.

The quantitative evaluation of the corrosion effects on the micro-scale consisted of determining the micromechanical properties of SWNTs on Ti-13Zr-13Nb alloy before and after the NSS test (Figure 14).

For comparative purposes, the non-anodized Ti-13Zr-13Nb alloy was also tested. The average Vickers microhardness strongly depends on the anodizing voltage. It takes a value of 271(12) for the non-anodized substrate compared to 65(8) for the Ti-13Zr-13Nb alloy anodized at 35 V for 120 min as determined before the NSS test. The HV_0.1_ value drops sharply for the sample anodized at 10 V, and for samples with the SWNTs layer produced at an anodizing voltage from 15 to 35 V, it takes similar values. After the NSS test, slightly lower HV_0.1_ values are observed than the HV_0.1_ values obtained before the NSS test. The difference in microhardness for SWNTs before and after the NSS test decreases with increasing anodizing voltage. Such changes in microhardness are related to the thickness of the oxide layer on the Ti-13Zr-13Nb alloy substrate and its surface morphology (Figure 4). It has been reported in the literature that the larger the diameter of the oxide nanotubes on titanium, the smaller the number of nanotubular structures that carry the load in the area of contact between the tested samples and the diamond indenter, as well as the easier the nanotubes deform and break [55]. From an application point of view, the SWNTs can compensate for the high hardness defect of the Ti-13Zr-13Nb alloy used as bone plates, avoid implant–bone stress mismatch, and reduce “stress shielding”.

It is worth noting that a direct relationship does not practically occur between the corrosion resistance to NSS and the corrosion resistance to other corrosive environments. The obtained results cannot be used for comparative testing to rank different materials relative to each other with respect to corrosion resistance or to predict the long-term corrosion resistance of the tested material. The NSS corrosion test can control the quality of a product or a given technological process. The NSS test is useful for detecting discontinuities, such as pores and other defects, in protective coatings [44]. In this work, the NSS test was used for a preliminary assessment of the applicability of newly developed SWNTs as permanent corrosion protection on the Ti-13Zr-13Nb alloy. The obtained results of the NSS test reveal the high surface quality of the Ti-13Zr-13Nb alloy subjected to anodizing under the proposed conditions. Both the high resistance of the produced SWNTs to the aggressive salt spray and their stable micromechanical properties were confirmed.

The main implications of the obtained results concern the derived linear equations describing the dependence between the outer and inner diameters of the oxide nanotubes and the applied anodizing voltage, which can be used in the future to produce tailored oxide nanotubes with assumed geometrical dimensions. Based on the obtained results, it can be possible to personalize the dose of the drug implemented on the surface of an implant made of Ti-13Zr-13Nb alloy with a drug carrier in the form of third-generation oxide nanotubes with the assumed morphological parameters. The developed Ti-13Zr-13Nb/SWNTs system with an increased total surface area can not only be used as a potential carrier of drugs that inhibit local infections and inflammations or support bone healing, including in metastatic bone cancer, but also can accelerate the osseointegration process due to the optimal nanotubular oxide structure.

## 4. Conclusions

The third-generation SWNTs were successfully produced for the first time on the biomedical Ti-13Zr-13Nb alloy by anodizing in 1 M C_2_H_6_O_2_ + 4 wt% NH_4_F electrolyte at U of 15–35 V for 120 min. No nanotubular oxide structures were formed at anodizing voltages of 5 and 10 V. The morphological parameters of the obtained SWNTs were functions of the anodizing voltage. With increasing anodizing voltage in the range of applied voltages, the outer diameter changed from 104(13) nm to 230(30) nm, whereas the inner diameter varied from 39(5) to 93(13) nm. The length of the SWNTs changed from 15.64(71) μm to 167.52(60) μm. The novelty of this work is the derivation of a linear dependence between the outer and inner diameters of the SWNTs, and the applied anodizing voltage was found.

The electronic properties of the obtained SWNTs studied by the SKP method depended on the anodizing voltage. Statistical analysis of the contact potential difference maps revealed that quantitative parameters as the arithmetic mean and root mean square of height irregularities increased with the oxidation voltage in the range of 5–35 V from −0.729 to −0.543 V and from 21.49 to 28.47 mV, respectively. The Ti-13Zr-13Nb alloy before anodizing was characterized by the smallest values of the contact potential difference, which proves the lowest stability among the tested materials.

The geometric structure of the Ti-13Zr-13Nb alloy after anodizing showed that the basic surface texture parameters Ra, Rz, Rp, and Rv are exponential functions of the oxidation voltage. The lowest Ra of 0.10 µm for the substrate before oxidation proved its smooth surface. The Ra of 1.07–2.73 was determined for the porous SWNTs surface obtained at 25–35 V, indicating the micro-rough surface for which the Ra belonged to the most suitable Ra for biomedical applications from 1 to 3 μm [6,9].

An assessment of corrosion resistance of the Ti-13Zr-13Nb alloy before and after anodizing to the effect of NSS on the macro scale showed the high quality of the obtained SWNTs. The quantitative evaluation of the impact of NSS on the micro-scale was based on the microhardness measurements before and after accelerated corrosion tests in a salt chamber. Before the NSS test, the average Vickers microhardness decreased with increasing anodizing voltage from 271(12) for the non-anodized substrate to 65(8) for the Ti-13Zr-13Nb alloy anodized at 35 V for 120 min. Only slightly lower HV_0.1_ was determined after the NSS test, confirming the high corrosion resistance of the developed SWNTs.

## Figures and Tables

**Table 1:** Salt spray chamber operating parameters in the NSS test according to ISO 9227:2017 [44].

Parameter	Value
NaCl concentration (collected solution)	52.4(6) g L^−1^
pH (collected solution)	6.6(1)
test temperature	34.8(7) °C
test time	168.0(1) h
average collection rate for a horizontal collecting area of 80 cm^2^	1.4(1) mL h^−1^

## Data Availability

Not applicable.
